# Effects of the Sequence of Isocaloric Meals with Different Protein Contents on Plasma Biochemical Indexes in Pigs

**DOI:** 10.1371/journal.pone.0125640

**Published:** 2015-08-21

**Authors:** Chunyan Xie, Xin Wu, Jun Li, Zhiyong Fan, Cimin Long, Hongnan Liu, Patrick Christian Even, Francois Blachier, Yulong Yin

**Affiliations:** 1 Hunan Provincial Engineering Research Center of Healthy Livestock, Key Laboratory of Agro-ecological Processes in Subtropical Region, Institute of Subtropical Agriculture, Chinese Academy of Sciences, Changsha, Hunan, 410125, China; 2 College of Animal Science and Technology, Hunan Agricultural University, Changsha, 410128, China; 3 School of Life Sciences, Hunan Normal University, Changsha, 41008, China; 4 INRA/AgroParisTech, UMR 914 Nutrition Physiology and Ingestive Behavior, Paris, France; CNRS, University of Strasbourg, FRANCE

## Abstract

Nutrient composition and pattern of food intake may play a significant role in weight gain. The aim of this study was to document the effects of a daily 3-meal pattern with isocaloric diets containing different dietary protein contents on growth performance and different plasma biochemical indexes including amino acid plasma concentration in castrated male pigs. Then, 21 DLY (Duroc×Landrace×Yorkshire) pigs aged 60 days were assigned randomly into 3 groups: a control group (crude protein, CP 18.1%), a group receiving high then basal and then low CP meals (High-Low group) and a group receiving low then basal and then high CP meal (Low-High group) for 40 days with pigs being feed-restricted. On day 40, after 12 h fasting, blood samples were obtained for analysis. The results showed that the insulin/glucagon ratio was lower in the High-Low group (P<0.05) when compared with the control group. Compared with the control group, the average daily gain of pigs from the High-Low group increased by 14.10% (P = 0.046). Compared with the control group, serum gamma-glutamyl transferase (GGT) decreased significantly (P<0.05) in both the High-Low and Low-High groups. Plasma concentrations of branched-chain amino acids (BCAA: valine, isoleucine and leucine) increased in the Low-High group (P<0.05) when compared with the control group; and plasma methionine and serine decreased in both the two experimental groups (P<0.05). Compared with the High-Low group, all the BCAA increased significantly (P<0.05) in the Low-High group. These findings suggest that the sequence and quantity of alimentary protein intake affect the insulin/glucagon ratio, as well as amino acid concentrations including BCAA, methionine and serine. It is proposed that meal pattern with pigs receiving high then basal and then low CP meals daily may help to improve the weight gain of pigs.

## Introduction

Pattern of nutrient consumption may reset peripheral circadian clocks and the related metabolic processes [1]. Dietary proteins are believed to participate significantly in the control of blood glucose levels. High-protein (HP) diet not only affects glucose homeostasis, but also may modify the metabolism in peripheral tissues [2]. Plasma amino acid concentrations which largely depend on the food ingested is the net result of protein digestion, amino acid and oligopeptide intestinal absorption, utilization of amino acid in anabolic pathways, release of amino acids from protein etc. [3,4]. In a recent report, it has been reported that a high protein meal given in the evening (40% of energy as protein) significantly increases the plasma free amino acids (PFAA) concentration measured on the next morning, thus even more than 12 hours after the meal [5].

All aspects of physiology, including sleep-wake cycles, cardiovascular activity, endocrine system, body temperature, renal activity, gastrointestinal tract motility, and metabolism, are influenced by the circadian clock [6]. Genome-wide circadian expression profiling studies have uncovered potential connections between circadian clocks and many aspects of metabolism, including energy, carbohydrate, amino acid, lipid, and protein metabolism [7]. Diets high in fat or sugar have been shown to alter circadian clock function [8]. Recent studies have linked energy homeostasis to the circadian clock at the behavioral, physiological, and molecular levels [9–11], emphasizing that certain nutrients and the timing of food intake and pattern of meals may play a significant role in weight gain [12].

This represents a paradigm shift in pig feeding, since the optimal dietary nutrient level is more and more considered as a dynamic process. Hence, we made the working hypothesis that a daily 3-meal pattern with different dietary protein contents may affect biochemical circulating parameters as well as body physiology and metabolism. Blood metabolite concentrations may help in interpretating some metabolic changes associated with different dietary modifications. Thus, the present study reports on the effects of feeding a daily 3-meal pattern with different dietary protein contents daily on several parameters including blood insulin, glucagon, and AA concentrations in the growing castrated male pigs.

## Materials and Methods

### Animals and experimental design

Twenty-one DLY (Duroc×Landrace×Yorkshire) barrows aged 60 days were obtained from Hunan New Wellful Co., Ltd. (Changsha city, 410005, China) [13]. The pigs were randomly distributed in 3 experimental groups according to the average body weight (BW = 20.80±1.74 kg, n = 21): control group, High-Low and Low-High groups, with 7 animals in each experimental groups. The pigs were housed in individual pens[14]. The barrows in the control group were fed with control diet (crude protein (CP), 18.1%) at 08:00, 13:00 and 18:00h. High-Low group of pigs was fed with high-CP diet (CP, 21.0%) at 08:00h, control diet at 13:00h and low-CP diet (CP, 15.3%) at 18:00h, while the Low-High group of pigs was fed with low-CP diet at 08:00h, control diet at 13:00h and high-CP diet at 18:00h. The pigs were feed restricted, and daily food amount was adjusted to 4.0% of BW and was given to pigs in 3 meals each day, each time with about one third of the total amount, and equal feed was given in the morning and evening for each group.

The nutrients were adequate for pigs and met the NRC-recommended requirement within the weight range used in the present study (NRC, 1998). The control diet was balanced on the calculated content of digestible energy (DE) (13.72 MJ/kg), CP (181.1 g/kg), apparent ileal digestible limiting amino acids (Lys, Met+Cys, Thr, Trp), Ca and P (Table 1). The control diet, high-protein and low-protein diets were composed as indicated in Table 1.

**Table 1:** Composition of the pig diets (as-fed basis).

Ingredients	High-Protein diet	Control diet	Low-Protein diet
**Corn (8% CP; < 13% H2O)**	54.6	63.5	72.4
**Soybean expanded (43% CP)**	29.00	21.27	13.54
**Enzymatically decomposed soybean meal (CP 48%)**	8.35	7.50	6.65
**L-lysine98%**	0.00	0.20	0.40
**L-methionine**	0.00	0.03	0.06
**L-threonine**	0.05	0.10	0.15
**Soybean oil**	4.00	3.40	2.80
**Premix** ^**1**^	4.00	4.00	4.00
**Total**	100.00	100.00	100.00
**Nutrient levels** ** **(%)**			
**CP**	21.04	18.11	15.29
**Lysine**	1.06	1.02	0.99
**Methionine**	0.33	0.32	0.31
**Methionine+Cystine**	0.67	0.62	0.57
**Threonine**	0.88	0.79	0.70
**Calcium**	0.58	0.60	0.55
**Total Phosphorus**	0.59	0.62	0.56
**Available Phosphorus**	0.41	0.42	0.39
**Digestible energy (MJ/kg)**	13.68	13.72	13.77

^1^ Providing the following per kg diet (‰): polymineral 25; carnitine 0.5; mixed vitamin 11.5; gourmet power 2.5; antioxidant 5; mildew preventive 12.5; Fe2(SO3)3 5; MgSO3 10; Cr 5; P-Ca 285; limestone 300; chaff 91.5; NaCl 75; feed carrier with zeolite powder. CP is Crude protein.

**nutrient level as calculated value.

All animals had free access to drinking water. The dietary intervention lasted 40 d.

### Samples collection

Body weights of individual pigs were measured immediately before feeding at the beginning and end of the trial[15]. On day 40, after 12 h fasting, the pigs were immobilized and then blood samples were collected by venipuncture from the venous sinus into two tubes: heparinized tubes and non-heparinized tubes[16]. The plasma was obtained by centrifugation at 3,000×*g* for 10 min at 4°C and then stored immediately at –80°C until assayed [13,17].

Feed intake and average daily gain (ADG) were recorded, and feed intake/ADG (F/G) was calculated for all pigs[18].

No animals were sacrificed in this study. This study was performed in accordance with the Chinese guidelines for animal welfare and approved by the Animal Care and Use Committee of the Institute of Subtropical Agriculture, the Chinese Academy of Sciences[19].

### Serum biochemical indices

An Automated Biochemistry Analyzer (Synchron CX Pro, Beckman Coulter, Fullerton, CA, USA) was used to determine the concentrations of serum glucose, urea nitrogen, gamma-glutamyl transferase (GGT), alanine aminotransferase (ALT), aspartate aminotransferase (AST) activities, total protein according to the commercial kits and manufacturer’s instructions [20]. All the kits were purchased from Beijing Chemlin Biotech Co., Ltd (Beijing, China).

### Serum hormone analyses

Serum insulin and glucagon were determined by radioimmunoassay according to corresponding reagent kit manufacturer’s instructions (China Institute of Atomic Energy, Beijing, China) [21].

### Determination of amino acids in plasma

Plasma (0.5 mL) was deproteinized with 0.5 mL of 1.5 mM HClO_4_, followed by addition of 0.25 ml of 2 M K_2_CO_3_[22]. The neutralized extract was analyzed for amino acids using high-performance liquid chromatography in Beijing Aminolabs (Beijing, China). This method involved the precolumn derivatization of amino acids with *o*-phthaldialdehyde and fluorescence detection[23]. Amino acids in samples were quantified on the basis of known amounts of standards (Sigma Chemicals, St. Louis, MO, USA).

### Statistical analysis

Data are presented as the mean ± SEM obtained from triplicate experiment. Differences between mean values of multiple groups were analyzed by one-way analysis of variance (ANOVA) followed by Post-hoc Tukey tests for multiple comparisons when interactions were significant using SPSS 13.0[24]. Differences between experimental groups were considered significant for P less than 0.05.

## Results

### Growth performance

The effects of pattern of consumption of diets with different protein contents daily on body weigh are shown in Table 2. The results showed that, compared with the control group, the ADG of pigs from the High-Low group increased by 14.1% (P = 0.046).

**Table 2:** Effects of the daily 3-meal pattern with different dietary protein contents on growth performance of growing pigs.

Items	Control group	High-Low group	Low-High group	P value
**Initial BW (kg)**	20.91±0.56	20.87±0.69	20.64±0.79	0.95
**Daily feed intake (g)**	1313.64±8.96	1324.32±14.05	1309.04±14.11	0.69
**Daily gain (g)**	528.13±21.41^b^	606.79±25.32^a^	546.07±21.39^a^ ^b^	0.048
**Feed/Gain**	2.46±0.06	2.15±0.10	2.41±0.09	0.074

Values are expressed as mean ± SEM, n = 7 pigs in each experimental groups.

^a,b^ indicate that means with different letters are significantly different (P<0.05).

Compared with the control and Low-High groups, F/G in the high-low group was not significantly different but tended to modestly decrease (0.05 < P < 0.1) by 12.3% and 10.5%, respectively (Table 2).

### Serum biochemical indices

Serum biochemical indices are shown in Table 3. Compared with the control group, serum glucose concentration from the High-Low and Low-High groups was not significantly different but tended to modestly increased by 6.2% and 10.02%, respectively. Serum GGT decreased significantly (P < 0.05) in both High-Low and Low-High groups, compared with the control group (Table 3).

**Table 3:** Effects of the daily 3-meal pattern with different dietary protein contents on plasma biochemical indexes of pigs.

Items	Control group	High-Low group	Low-High group	P value
Glucose	4.39±0.14	4.67±0.10	4.83±0.43	0.455
Total protein	64.02±2.60	64.02±2.37	64.65±1.47	0.973
Gamma-glutamyl transferase (GGT) (U/L)	79.52±18.04^a^	41.65±6.71^b^	40.6±2.41^b^	0.033
Alanine aminotransferase (ALT) (U/L)	59.33±4.16	59.33±5.16	50.6±7.03	0.519
Aspartate aminotransferase (AST) (U/L)	46.20±2.84	56.33±8.97	44.83±3.2	0.351
Lactate Dehydrogenase (U/L)	524.2±52.79^a^ ^b^	599.6±95.22^a^	372.7±20.67^b^	0.048
Urea (mmol/L)	4.32±0.29	5.19±0.56	5.04±0.48	0.361

Values are expressed as mean ± SEM, n = 7 pigs in each experimental groups.

^a,b^ indicate that means with different letters are significantly different (p<0.05)

Compared with the Low-High group, serum LDH in the High-Low group was lower (P < 0.05). Compared with the control group, serum urea concentration was not significantly different in the High-Low group and Low-High group but tended to increase by 20.14% and 16.67% (Table 3).

### Serum insulin and glucagon

Serum hormone concentrations are shown in Table 4. No difference was observed for insulin, but serum glucagon was higher in the High-Low and Low-High groups, the difference being significant however only in the High-Low group. Accordingly, the insulin/glucagon ratio was lower in both the High-Low and the Low-High groups than in the control one (P < 0.05) (Table 4). No difference was observed in the serum insulin concentrations after a High-Low or Low-High meal intake.

**Table 4:** Effects of the daily 3-meal pattern with different dietary protein contents on serum insulin and glucagon.

Items	Control group	High-Low group	Low-High group	P value
**Insulin (mU/L)**	5.11±0.19	4.75±0.17	5.02±0.15	0.323
**Glucagon (ng/L)**	270.57±14.08^b^	326.9±12.31^a^	313.9±9.36^a^ ^b^	0.014
**Insulin/Glucagon**	0.019±0.0007^a^	0.015±0.0005^b^	0.016±0.0006^b^	0.0001

Values are expressed as mean ± SEM, n = 7 pigs in each experimental groups.

^a,b^ indicate that means with different letters are significantly different (P < 0.05).

### Plasma amino acids

**Fig 1:**
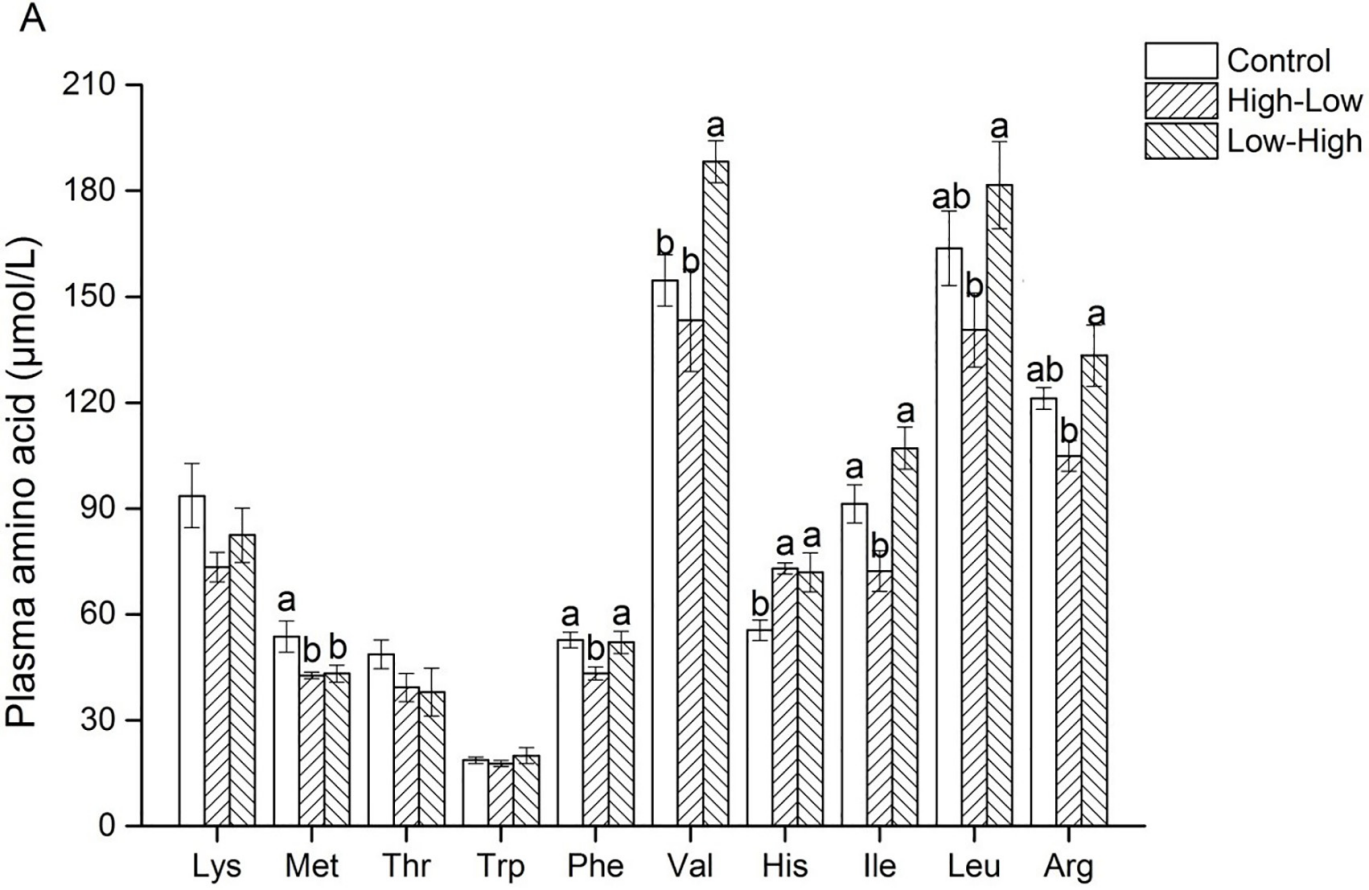
Effects of the daily 3-meal pattern with different dietary protein contents on plasma essential amino acids in pigs. N = 7.^a-b^Means within a row with different letters differ (P<0.05). Lys, L-lysine; Met, L-methionine; Thr, L-threonine; Trp, L-tryptophan; Phe, L-phenylalanine; His, L-histidine; Val, L-valine; Ile, L-isoleucine; Leu, L-leucine; Arg, L-arginine.

For the essential amino acids, compared with the control group, plasma valine was higher in the Low-High group (P < 0.05), plasma leucine was lower in the High-Low group (P < 0.05) and plasma methionine was lower by 20.4% (P < 0.05) and 19.4% (P < 0.05) respectively in the High-Low and Low-High (Fig 1).

**Fig 2:**
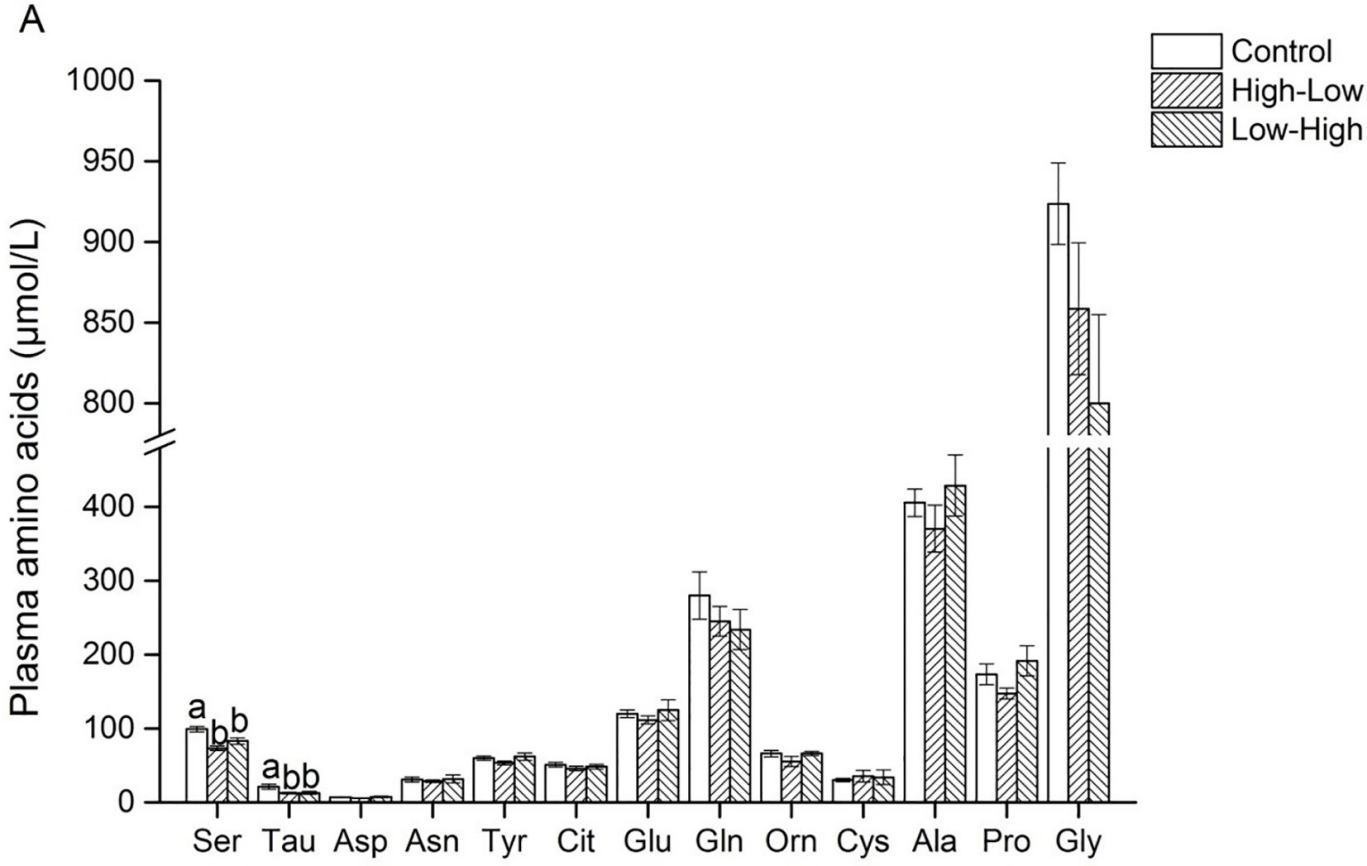
Effects of the daily 3-meal pattern with different dietary protein contents on plasma non-essential amino acids in pigs. n = 7 pigs. Bars with different letter are significantly different (p<0.05). Ser, L-serine; Tau, taurine; Asp, L-aspartic acid; Asn, L-asparagine; Tyr, L-tyrosine; Cit, L-citrulline; Glu, L-glutamic acid; Gln, L-glutamine; Orn, L-Ornithine; Cys, L-cystine; Ala, L-alanine; Pro, L-proline; Gly, glycine.

For the non-essential amino acids, plasma serine and taurine were lower in the two experimental groups (P < 0.05) (Fig 2). Differences were also observed between the two experimental groups; plasma concentrations of BCAA (valine, isoleucine and leucine) were larger and plasma arginine was lower in the Low-High group than in the High-Low group.

**Fig 3:**
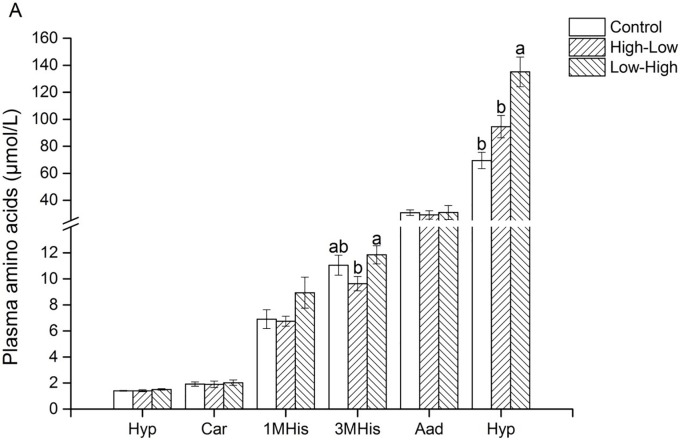
Effects of the daily 3-meal pattern with different dietary protein contents on plasma metabolic markers of amino acids of pigs. n = 7 pigs; ^a,b^ indicate that bars with different letter are significantly different (p<0.05). Hyp, hydroxy-L-proline; Car, L-carnosine; MHis, Methyl-L-histidine; Aad, L-α-amino-n-butyric acid; Hyp, L-homocystine.

Similarly, according to the dietary pattern, diets containing an equivalent amount of nutrients had different effects on several indicators of the metabolism of amino acids (Fig 3). Indeed, plasma 3-Methyl-L-histidine (MHis) was 23.1% higher (P < 0.05) and plasma homocysteine (Hyp) was x% higher (P < 0.05) in the Low-High than in the High-Low group.

## Discussion

Circadian clock plays a crucial role in the regulation of numerous physiological processes and the beneficial versus deleterious effects of a given diet may be partly related to the sequence of dietary intake in the daytime [12]. Circadian rhythms are generated by one of the most ubiquitous timing systems related to numerous behavioral, physiological, cellular and molecular processes that are controlled by an endogenous clock which depends on environmental factors including light, food and stress [25]. Particularly, circadian control of metabolism has been widely studied. The circadian clock controls food intake and energy homeostasis by regulating the expression and/or activity of enzymes involved in the metabolism of numerous compounds including cholesterol, amino acid, lipid, glycogen, and glucose, as well as the secretion of many hormones involved in metabolism, such as insulin, glucagon, leptin, and ghrelin hormones that exhibit circadian oscillation [26]. In addition, diet-induced thermogenesis is known to show circadian variation, with the highest response in the morning and the lowest in the evening in humans [27]. The body’s nutrient digestion, absorption and utilization may differ according to the different periods of time (morning, noon and night), and according to nutritional needs. Thus, eating habits may contribute to body weight changes. The results obtained in the present study, which are primarily related to optimal pig growth in an agronomic context, may be also relevant in terms of human nutrition. Pigs, like humans, are omnivorous animal and make individual meals [28]. Besides their importance in livestock production, pigs are of particular biomedical interest because of their phylogenetic relation to humans, and sharing many physiological similarities with humans, offers several specific advantages (when compared to non-human primates). Castrated pigs can avoid unwanted or uncontrolled reproduction, and are easy for management. Interestingly, ADG in response to the High-Low feeding method was higher than that measured in to the control group and, since caloric intake was an equivalent food efficiency also tended to be increased. In a recent study, modifications of the daily-phase feeding system did not significantly modify body weight but reduced N excretion by 12% (P < 0.01) [29]. Whatever the dietary pattern of the nutrient consumption (including protein consumption) during the day, some homeostasis of the circulating and tissue nutrient concentration has to be maintained. Glucose being the primary fuel source for most cells, it is for instance important for blood glucose levels to remain relatively steady. As a matter of fact, blood glucose levels may be affected by the timing of meals and snacks. Experimental data suggest that breakfast frequency and quality may be related in a causal way to appetite and blood sugar control [30]. High-CP diets have been reported to have positive effects on glucose homoeostasis in rats [31] and humans [32]. In the present study, serum glucose concentration from the High-Low and Low-High groups was not significantly different but tended to modestly increased by 6.2% and 10.02% when compared with the control group.

Transamination in cells and tissues is accomplished by enzymes called transaminases or aminotransferases. This process is an important step in the synthesis of some non-essential amino acids and urea synthesis. The activities of enzymes as well as some endocrine responses are related to these metabolic pathways and are also known to be modified by time-restricted feeding [33]. Elevated levels of serum GGT have been found to predict the risk of developmenting of type 2 diabetes in adults and children [34]. In the present study, daily 3-meal pattern with different dietary protein contents affected fasting plasma GGT significantly, suggesting that the transamination system via urea-cycle is affected in the liver at 12 hours after the ingestion of a variable nutritional levels during the day. Further experiments are needed to confirm this hypothesis. However, our results may indicate that daily 3-meal pattern with different dietary protein contents may affect amino acid requirements and metabolic availability. It has been reported recently that elevated ALT levels, within the normal physiological range, are associated with unfavorable nocturnal glucose profiles in Chinese subjects with normal glucose regulation [35].

It is noteworthy that behaviors and physiological activities in animals display circadian rhythms, allowing the organisms to anticipate and prepare for the diurnal changes in the living environment [36]. Plasma amino acid profiles also exhibit a circadian rhythm [37,38]. Amino acids, normally supplied by dietary protein or by endogenous production, are necessary for anabolic metabolism and signaling purposes including the biosynthesis of polypeptides and proteins, and the synthesis of nucleotides. In the present study, we found that among amino acids, the most significant differences between experimental groups were observed for plasma BCAA concentrations and for some urea-cycle related compounds [5]. Plasma concentrations of the BCAA (leucine, isoleucine, and valine) are more prominently affected than the concentrations of other amino acids by changes in dietary-caloric, protein, fat, and carbohydrate-intake in man [39]. In our study, it is interesting to note that plasma BCAA concentrations significantly increased in pigs receiving the Low-High diet in comparison with what is observed in the High-Low group; and tended to be higher when compared with the control group. Among BCAA, leucine has been shown in animal models to promote nitrogen retention and protein synthesis as well as inhibition of protein degradation [40,41]. This finding is consistent with previous observations indicating that a high protein meal in the evening significantly increased the plasma BCAA concentration on the next morning, even more than 12 hours after the meal [5], demonstrating that varying the protein content in the meal pattern with different dietary protein contents may affect the use of some essential amino acids. These changes appear partly related to the changes of transaminating enzyme activities, even if possible causal links between these parameters remain to be demonstrated.

It is well known that proteins have a notable impact on glucose homeostasis mechanisms, predominantly through their effects on insulin, incretins, gluconeogenesis, and gastric emptying [42]. Insulin and glucagon are two important hormones for blood glucose regulation. Protein, but not carbohydrate, lead to increased glucagon secretion following a meal [43,44]. According to classical studies, administration of carbohydrate has a protein-sparing effect in the fasting subject [45,46].Protein induces an increase in insulin concentrations when ingested in combination with carbohydrate. It is reported that a mixture of wheat protein hydrolysate, free leucine, phenylalanine, and carbohydrate can be applied as a nutritional supplement to strongly elevate insulin concentrations [47]. The insulin/glucagon ratio determines whether glucose uptake or output production [48]. It has been reported that the insulin/glucagon ratio is highest during daylight on a high protein diet and in late night on a control diet [49]. In the present study, it is interesting to note that in the High-Low group, we found no effect on insulin, but higher plasma glucagon than in the control group (this tendency was also observed in the Low-High group). In addition the insulin to glucagon ration was very significantly decreased in both experimental groups. This implies that the varying the protein content in the meal affected plasma glucagon and increased the drive for glucose production in the fasting state. Serum glucagon concentration has been significantly correlated with protein intake [50]. Feeding a high-protein diet resulted within 3 hours in an increase in the concentration of insulin (+100%) and glucagon (+220%) [48].

Previous studies have shown that the glucagon response to feeding with protein depends on the increase in plasma amino acid concentrations [51]. It has been reported that the insulin response is closely related to the increase in plasma amino acids, especially leucine, isoleucine, valine, phenylalanine and arginine, regardless of the rate of gastric emptying; while the glucagon response is linearly related to the increase in plasma amino acids, regardless of the rate of gastric emptying or meal composition. Among the plasma amino acids, tyrosine and methionine have been closely related to the plasma glucagon response, and it has been shown that the glucagon response to feeding with protein depends on the increase in plasma amino acid concentrations [51].

## Conclusion

The findings reported in the present study strongly suggest that the varying the protein content in the meal affects the insulin/glucagon ratio, and amino acids including BCAA, methionine and serine in pigs. In this study, it was found that the varying meal protein content affects circulating biochemical parameters, which may impact the growth performance of pigs, and then may be useful for pig production. In practice, this study suggests that the High-Low feeding procedure improved growth and feed efficiency.
